# Editorials

**Published:** 1868-05-15

**Authors:** 


### *EDITORIAL.*

### *Apologetic.*

The present number of the JOURNAL is delayed a few days in expectation of being able to send out with it the Annual Announcement of Rush Medical College. Circumstances, however, rendered it necessary to put that over until the 1st of June number, which will be issued several days in advance of its date.

### *Students' Number.*

Under date of June 1st, we shall issue a Students' number of the JOURNAL. Of that number we shall send out *twelve thousand* copies. Aside from the Announcement, it will contain foreign and domestic correspondence, with several communications of great interest. The forms will be stereotyped, and we shall send as many more as we can obtain names to address. All our friends will oblige us by sending names of physicians, medical students and druggists.

### *Variety.*

Hereafter the JOURNAL will contain a variety which heretofore the length of several elaborate articles published has prevented. The exhaustive thesis of Dr. Bosworth will be completed in the number for June 15th. We do not hesitate to say that it alone is worth the annual subscription to the JOURNAL.

### *Communications on File.*

H. Webster Jones, M.D., Observations on Treatment of Sterility.

A. J. Miller, M.D., Shoulder Presentations.

E. R. Travers, M.D., " "

T. R. Wagoner, M.D., " "

N. Teall, M.D., " "

L. T. Strother, M.D., Extra Uterine Fœtation.

Wm. L. Coe, M.D., Mistaken Diagnosis.

A. Catron, M.D., Cerebro Spinal Meningitis.

H. D. Garrison, Fluid Extracts.

H. Wanzer, M.D., Placenta Prævia.

A. J. Baxter, M.D., Calabar Bean in Tetanus.

Cook County Hospital, Albuminuria Mitral Insufficiency.

T. O. Edwards, M.D., "Nothing new under the Sun."

Walter Hay, M.D., Translations of papers read before the Royal Academy, Paris, continued. Also a large number of miscellaneous papers which the Editor has not yet had time to look over.

### *Blood Corpuscles.*

The beautiful and suggestive discovery, by Prof. Freer, described in the first article of this JOURNAL, will attract large attention. We are able to confirm his observations by personal investigation. His improved method gives results of the most remarkable character in conditions of the blood varied by disease.

### *Medical Education.*

Our friends of the Cincinnati *Lancet and Observer*, furnish the profession an article on this somewhat hackneyed subject which we shall not scruple to transfer to our pages, *bodily*, so soon as space will permit. The Apostle therein takes the new (public) *role* of the "Phantom in Black." Unhappily our copy of the *Lancet* was borrowed by a friend, who failed to return it in season for insertion of the article in the present number of the Chicago Medical Journal. Meanwhile we endorse its sentiments *ad unguem*.

### *It is Queer*

That medical gentlemen do not all of them see that medical science is developing mainly by *excision* not by *accretion*. That notably the most ignorant men who have, *ex gratia*, secured the diploma of M.D., are the most concerned about the *status* of the profession.

Many years ago we were impressed with the truth of a proposition by Dr. Wayland, of Brown University: "Let your *character* take care of your *reputation.*"

Legal enactments — the verdicts of juries and the *dicta* of big-wigged judges — are not worth, to the profession, the powder to blow them to Gehenna. If you wish to be respected, *be* respected. *Hoc opus, hic labor est!*

Brother Haller — *Vandal-ia* like, wishes this to be accomplished by resolution. He wishes (perhaps under the stimulus of an honorary degree conferred by the APOSTLE) to have this done by a jury of inquest to be appointed by some dirty politician, accidentally governor.

In our observation the most ignorant men clamor loudest for "Medical Reform." Educated medical gentlemen scorn the splints and braces of temporary political demogogues.

Witness Michigan, with its *big* Medical College, gone to hopeless ruin under the domination of political hacks.

A man, intimately connected with the sewerage of Chicago, started a Medical College. To-day, because respectable medical gentlemen are educated to believe that respectability is the offspring of real worth, they are denounced in the circles of petty intrigue as unworthy of trust. The rattlesnakes lie in wait for them, slimy and secret, but *educated* medical men know that their fangs have been removed. We repeat, to-day, medical men stand highest, not by legal enactments — not by trades-union devices — not by pitiful advertising schemes, but by real merit, by high attainment.

Let the Apostle continue his jeremiads, and his disciples imagine a vain thing — the profession to-day is respected because it is RESPECTABLE.

Will the State Medical Society enact impotence into virility? If so, let it understand that there is still truth in the old maxim *ex nihilo, nihil fit.*

The following gentlemen have been acting in coöperation with members of the Faculty of Rush Medical College, as

### *Lecturers and Instructors*

during the Spring Course and series of instruction in the interim of the Annual Sessions the preceding year:

WELLS R. MARSH, M.D., *Princ. and Prac. Med. and Dispensary Physician*, 59 W. Randolph Street.

JOHN E. OWENS, M.D., *Surgery and Venereal Diseases*, St. Luke's Hospital and 112 Randolph Street.

WM. C. LYMAN, M.D., *Surgery and Surgical Diagnosis*, U. S. Marine Hospital, cor. Wabash Avenue and Washington Street.

CURTIS T. FENN, M.D., *Obstetrics, etc.*, Cook County Hospital.

CHARLES T. PARKES, M.D., *Anatomy, etc.*, 43 N. Clark Street.

WM. C. HUNT, M.D., *Microscopy and Histology*, College.

I. N. DANFORTH, M.D., *Chemistry, etc.*, 43 S. Clark Street.

